# Anti-*Toxoplasma* activity of silver nanoparticles green synthesized with *Phoenix dactylifera* and *Ziziphus spina-christi* extracts which inhibits inflammation through liver regulation of cytokines in Balb/c mice

**DOI:** 10.1042/BSR20190379

**Published:** 2019-05-15

**Authors:** Reem A. Alajmi, Wafa A. AL-Megrin, Dina Metwally, Hind AL-Subaie, Nourah Altamrah, Ashraf M. Barakat, Ahmed E. Abdel Moneim, Tahani T. Al-Otaibi, Manal El-Khadragy

**Affiliations:** 1 Department of Zoology, College of Science, King Saud University, Riyadh, Saudi Arabia; Department of 2 Biology, Faculty of Science, Princess Nourah bint Abdulrahman University, Saudi Arabia; 3 Department of Parasitology, Faculty of Veterinary Medicine, Zagazig University, Zagazig, Egypt; 4 Department of Zoonotic Diseases, National Research Centre, Dokki, Giza, Egypt; 5 Department of Zoology and Entomology, Faculty of Science, Helwan University, Cairo, Egypt

**Keywords:** nanoparticles, Mice, Toxoplasma gondii

## Abstract

Toxoplasmosis constitutes a global infection caused by oblige intracellular apicomplexan protozoan parasite *Toxoplasma gondii*. Although often asymptomatic, infection can result in more severe, potentially life threatening symptoms particularly in immunocompromised individuals. The present study evaluated the anti-*Toxoplasma* effects in experimental animals of silver nanoparticles synthesized in combination with extracts of natural plants (*Phoenix dactylifera* and *Ziziphus spina-christi*) as an alternative method to standard sulfadiazine drug therapy. Liver functions estimated by and AST and ALT were significantly increased in *T. gondii*-infected mice compared with the control group as well as hepatic nitric oxide (NO), lipid peroxidation (LPO) levels and caused significant decrease in superoxide dismutase (SOD), catalase (CAT) and glutathione activities in the liver homogenates. Nanoparticles pretreatment prevented liver damage as determined by enzyme activity inhibition, in addition to significant inhibition of hepatic NO levels and significant elevation in liver SOD and CAT activities. Moreover, nanoparticle treatment significantly decreased hepatic LPO and NO concentrations and proinflammatory cytokines but significantly boosted the antioxidant enzyme activity of liver homogenate. In addition, histological examinations showed distinct alterations in the infected compared with untreated control groups. Conversely, nanoparticles pretreatment showed improvement in the histological features indicated by slight infiltration and fibrosis, minimal pleomorphism and less hepatocyte and degeneration. Furthermore, nanoparticles treatment induced a reduction in immunoreactivity to TGF-β and NF-κB in hepatic tissues. Therefore, the present study provides new insights into various natural plants that are used traditionally for the treatment of toxoplasmosis and other parasitic infections, which may be useful as alternative treatment option for *T. gondii* infections.

## Introduction

*Toxoplasma gondii* Nicolle and Manceaux, 1909 is protozoan parasite belonging to the family Sarcocystidae Poche, 1913, and constitutes one of the world’s furthermost common bloodsuckers [1]. It has an indirect life cycle through cat as a definitive host [2]. Toxoplasmosis is public because the parasite has stumpy host specificity and can infect a range of hosts, excluding nearly one-third of human population [3]. *Toxoplasma* infection can be mortal in pregnant or immunocompromised individuals [4], and has been associated with abortion, encephalitis and neonatal mortalities in such cases [5,6]. In healthy persons who are infected, the proliferation of the parasites is addressed by administered drugs and the immune system; however, cysts persist in all infected tissues, including the brain, so helping as a source for subsequent active parasite infection [7]. Hence, effective treatment are needed for all infected cells to avoid cyst formation [8]. Still, current treatment choices for patients with toxoplasmosis are restricted [9]. These comprise the usage of antibiotics and anti-malarial drugs, which often source to side effects, as well as rashes and bone marrow suppression [10,11]. So, toxoplasmosis characterizes a large global burden that is further improved by the shortcomings of the current therapeutic options [12]. These elements emphasize the urgent need for better anti-*Toxoplasma* drugs and/or new approaches in the treatment of toxoplasmosis.

An best anti-*Toxoplasma* drug would be non-toxic, potent and able to eradicate the latent infection [13]. As there is going up interest in using nanotechnology, some reports suggest that nanoparticles (NPs), for biomedical purposes, could form the bulk of future treatment strategies for different diseases [14]. Currently, nanoparticles are being used for numerous biomedical applications owing to their nanoscale proportions and other useful surface reactivity. In addition, NPs can produce free radicals that have the talent to kill infectious agents [15] with the minor size of NPs allowing them to membrane barriers leading to more reactivity [16]. Furthermore, nanoparticles could be gathered in tissues, thereby presenting a formidable platform to cysts in host tissues [11]. Of special interest for this purpose are the metal nanoparticles such as silver and gold, which exhibit anti-microbial [17], anti-parasitic [14], and other bioactivities as well as the selective prevention of some enzyme activities [18]. The versatility of metal NPs thus renders them a gorgeous choice to be sightseen further as anti-parasitic agents, mainly against toxoplasmosis [19].

Accordingly, the present study was aimed to evaluate the potential effects of silver NPs (AgNPs) as new anti-*T. gondii* agents via *in vivo* experimental infection models.

## Materials and methods

### Preparation of NPs

#### Preparation of date seeds (*Phoenix Dactylifera*)

Date seeds were dripped in water, then wash away to free any adhering date flesh, air-dried, and then further dried in an air oven at 60°C. The dried date palm seeds were broken into several pieces by grinding in a heavy-duty grinder to pass through 1–2 mm screens to produce date palm seed flour or date-pit powder and then kept in freezer at −20°C until used. For subsequent experiments, 5 g of date seed powder was soaked in 100 ml boiled distilled H_2_O overnight. The extract was filtered for preparation of the NPs.

#### Preparation of *Ziziphus spina-christi* (Nabka)

Nabka leaves were collected from Riyadh, Saudi Arabia. The plant identification was confirmed by the Department of Botany and Microbiology, College of Science, King Saud University, Riyadh, Saudi Arabia. The plant leaves were then dried and ground into powder, which was extracted with 70% methanol. In brief, the powder was mixed and incubated at 4°C for 24 h. *Ziziphus spina-christi* leaf extract (ZLE) was filtered and then dryness in vacuum evaporator (Heidolph Instruments, Schwabach, Germany). The residue was dissolved in distilled H_2_O and used in this experiment.

#### Preparation of AgNPs

A total of 3–5 ml of the resulting date seed extract were added to 0.1 mM/ml AgNO_3_ and was stirred at 45–50°C. To this, 5 g of nabka powder was added and forming AgNPs. UV-visible spectroscopy analysis was carried out at a wavelength range 190–1100 nm using a Perkin Elmer Lambda 25 UV-visible spectrometer (Beaconsfield, U.K.). The mean size of the silver NPs was analyzed using a Zetasizer (Nano series, HT Laser, ZEN 3600, Malvern Instruments, Malvern, U.K.), whereas transmission electron microscopy (TEM, JEM-1011, JEOL, Tokyo, Japan) was employed to determine particle shape, size and morphologies of synthesized AgNPs with an quickening voltage of 100 kV.

#### Sulphadiazine

Microionized powder was kindly supplied by National Organization for Drug and Research (NOFDR) B. No. B 239933AA, and 100 mg/tab were used. It was given orally at a dose of 100 mg/kg /days for 15 consecutive days for the infection model.

#### Experimental animals and handling

A total of 42 female Balb/c mice, 5–6 weeks old, approximately 25–30 g, were obtained from the laboratory Animal Breeding Council (King Saud University of Medical Science, Riyadh, Saudi Arabia). They were housed in a room under controlled temperature (24 ± 2°C), lighting (12-h light/dark cycle), and relative humidity 40–70% conditions. They received a standard diet and water *ad libitum*. All animals were handled according to recommendation of the Ethics Committee at King Saud University, Riyadh, Saudi Arabia.

#### Parasites and antigen preparations

The virulent RH strain of *T. gondii* (RH HXGPRT) was obtained from King Saud University of Medical Sciences, Riyadh, Saudi Arabia. Tachyzoites of this strain were collected by serial intra-peritoneal passages in BALB/c mice. Parasites (1 × 10^5^) were inoculated in mice, and after 72 h, tachyzoites were provided by repeated flushing of the peritoneal cavity by phosphate-buffered saline (PBS). Tachyzoites were then harvested and centrifuged at 200×*g* for approximately 5 min at room temperature. The supernatants were discarded and pellets enriched with parasite tachyzoites were recovered with PBS and used in the experiment.

#### Experimental design

The collected animals were divided into six groups each containing seven mice; the first group served as a normal non-infected negative control group, the second group was the infected non-treated positive control group, the third group was the post-infected group receiving oral 100 mg/kg sulfadiazine administered daily 2 weeks prior to infection, the fourth group was post-infected receiving 100 mg/kg AgNPs green synthesized with date palm seed and nabka extracts orally administrated daily for 2 weeks prior to infection, the fifth group was infected and then treated with sulfadiazine, and the sixth group was infected and then treated with AgNPs (date palm seeds and nabka) on the same day of infection.

#### Serum preparation

Blood samples were obtained by cardiac puncture into sterile vacuum tubes with and without anticoagulant (EDTA). Serum was separated by centrifugation at 3000 rpm for 20 min at 4°C and stored at −20°C until used for biochemical assays.

#### Survival study

Survival rate was monitored for 7 days from the beginning of the therapy until all mice died. The mean survival times of each study group were analyzed via Kaplan–Meier survival curve, which was drawn by Prism version 6.

### Biochemical analysis

#### Liver functions

Aspartate aminotransferase (AST) and alanine aminotransferase (ALT) activities in the serum were determined according to Reitman and Frankel [20]. This biochemical analysis was measured calorimetrically using a spectrophotometer and purchased kits provided by Randox Laboratories Co. (Crumlin, U.K.).

#### Oxidant/antioxidant assessment

Liver homogenates were used to determine lipid peroxidation (LPO) and nitrite/nitrate (nitric oxide, NO) measured using a biodiagnostic assay kits according to the methods of Ohkawa et al. [21] and Green et al. [22], respectively. Liver homogenates were used for the determination of glutathione (GSH) according to method of Ellman [23], superoxide dismutase (SOD) as described by Nishikimi et al. [24], and catalase (CAT) was assayed colorimetrically as described by Aebi [25].

#### Inflammation markers interferon-γ and TNF-α levels determination

Quantitative measurements of INF-γ (Cat. no. BMS606, ThermoFisher Scientific) and TNF-α (Cat. no. EZMTNFA, Millipore) levels were performed using enzyme-linked immunosorbent assay (ELISA) kits specified for mice according to the protocol provided with each kit.

#### Molecular analysis

Total RNA was isolated from liver tissue using an RNeasy Plus Minikit (Qiagen, Valencia, CA) following the manufacturer recommended protocol. RNA concentration and quality were determined spectrophotometrically by measuring absorbance at wavelengths of 260 and 280 nm. Total RNA (1 µg) and random primers were used for cDNA synthesis using RevertAid H Minus Reverse Transcriptase (Fermentas, Thermo Fisher Scientific Inc., Burlington, Ontario, Canada) according to the manufacture’s suggestions. For real-time polymerase chain reaction (PCR) analysis, the cDNA samples were run in triplicate. PCR amplification included non-template controls containing all reagents except cDNA. Real-time PCR was performed using Power SYBR Green (Life Technologies, Carlsbad, CA) and conducted on an Applied Biosystems 7500 Instrument (Foster City, CA). The typical thermal profile was 95°C for 3 min, followed by 40 cycles of 95°C for 15 s and 56°C for 30 s. After PCR amplification, the Δ*C*_t_ was calculated by subtraction of the β-actin *C*_t_ from each sample *C*_t_ using Applied Biosystems Step One™ Instrument software. The method of Pfaffl was used for data analysis. PCR primers for Sod2, and Cat genes were synthesized by Jena Bioscience GmbH (Jena, Germany). Primers were designed using Primer-Blast software from NCBI. As a reference gene, β-actin was used. The primer sets used were **SOD2** (S): 5′-GCC CAA ACC TAT CGT GTC CA-3′, (AS): 5′-AGG GAA CCC TAA ATG CTG CC-3′, **CAT** (S): 5′-CCG ACC AGG GCA TCA AAA-3′, (AS): 5′-GAG GCC ATA ATC CGG ATC TTC-3′, **iNOS** (S): 5′-CGA AAC GCT TCA CTT CCA A-3′, (AS): 5′-TGA GCC TAT ATT GCT GTG GCT-3′, **IL-1β** (S): 5′-TGC CAC CTT TTG ACA GTG ATG-3′, (AS): 5′-TTC TTG TGA CCC TGA GCG AC-3′, and **β-Actin** (S): 5′-GCA GGA GTA CGA TGA GTC CG-3′, (AS): 5′-ACG CAG CTC AGT AAC AGT CC-3′.

### Histopathological examination

#### Light microscopy study

Immediately after scarification, samples of the livers from each group were collected and fixed in 10% neutral formalin for 24 h and paraffin blocks were generated and routinely processed for light microscopy. Slices of 4–5 μm were obtained from the prepared blocks and stained with hematoxylin and eosin or Sirius Red. The preparations obtained were visualized using a Nikon microscope to evaluate the pathological changes.

#### Immunohistochemistry for the detection of transforming growth factor-β (TGF-β) and nuclear factor-κB (NF-κB)

Liver sections were deparaffinized and then boiled to unmask antigen sites, and the endogenous activity of peroxidase was quenched using 0.03% H_2_O_2_ in absolute methanol. Liver sections were incubated overnight at 4°C with 1:200 dilution of goat polyclonal anti-TGF-β or anti-NF-κB antibodies (Santa Cruz Biotechnology, Dallas, TX) in PBS. After removal of the unbound primary antibodies by rinsing with PBS, slides were incubated with 1:500 dilution of biotinylated anti-goat secondary antibody. Bound antibodies were detected with the Vectastain avidin biotinylated peroxidase complex ABC-kit (Vector Laboratories, Burlingame, CA) and the chromogen 3,3′ diaminobenzidine tetrachloride was used as a substrate. After appropriate washing in PBS, slides were counterstained with hematoxylin. All sections were incubated under the same conditions with the same concentration of antibodies and at the same time; therefore, the immunostaining was comparable among the different experimental groups.

### Statistical analysis

Results were expressed as the means ± standard error. Data for multiple variable comparisons were analyzed by one-way analysis of variance (ANOVA). For the comparison of significance between groups, Duncan’s test was used as a post hoc test according to the statistical package program SPSS ver. 22 (Chicago, IL). All *P*-values are two-sided and *P*<0.05 was considered as significant for all statistical analyses in this study.

## Results

### Characterization of AgNPs

**Figure 1:**
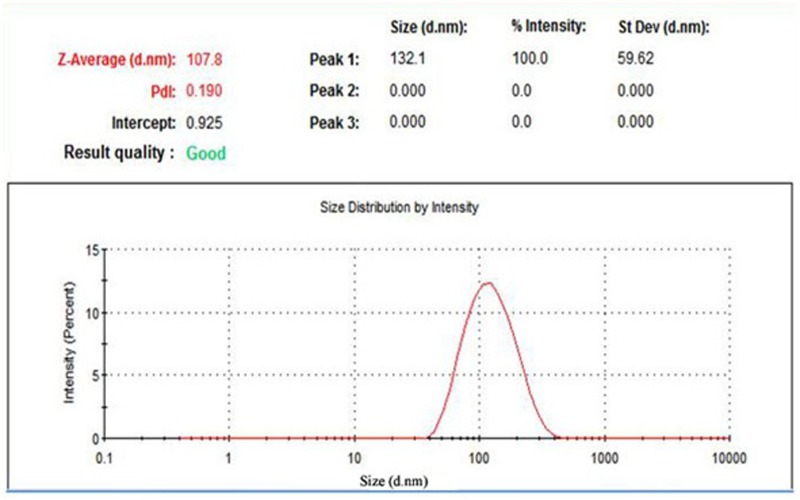
Size distribution of the formed AgNPs

**Figure 2:**
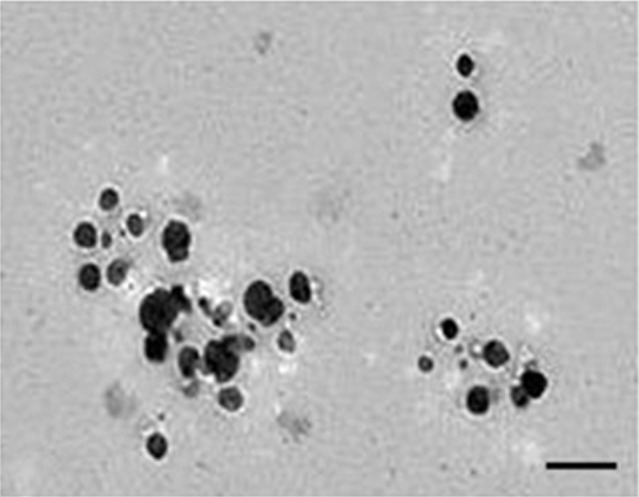
TEM micrographs showing the size of synthesized silver nanoparticles; scale bar is 50 nm

**Figure 3:**
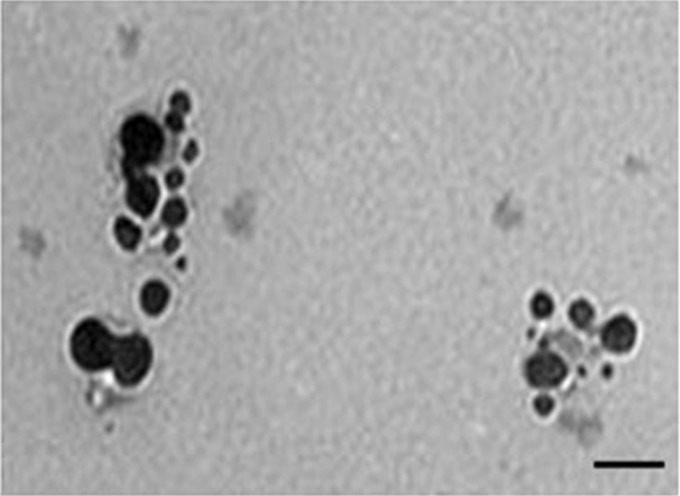
TEM micrographs showing the size of synthesized silver nanoparticles; scale bar is 50 nm

Dynamic light scattering (DLS) analysis of the formed AgNPs using the Zetasizer showed non-homogeneous AgNPs with a mean particle size of 200 nm that could be observed as the appearance of a single peak (Figure 1). TEM was used to visualize the size and shape of synthesized AgNPs produced via leaf extracts of *P. dactylifera* and *Z. spina-christi.* It is evident from Figures 2 and 3 that the majority of the synthesized particles were irregular in shape with some spherical NPs. The average size of the individual AgNPs was estimated at 20 nm. The maximum and minimum sizes of synthesized individual AgNPs were found to be 22.8 and 13.2 nm, respectively.

### Mean survival time

**Figure 4:**
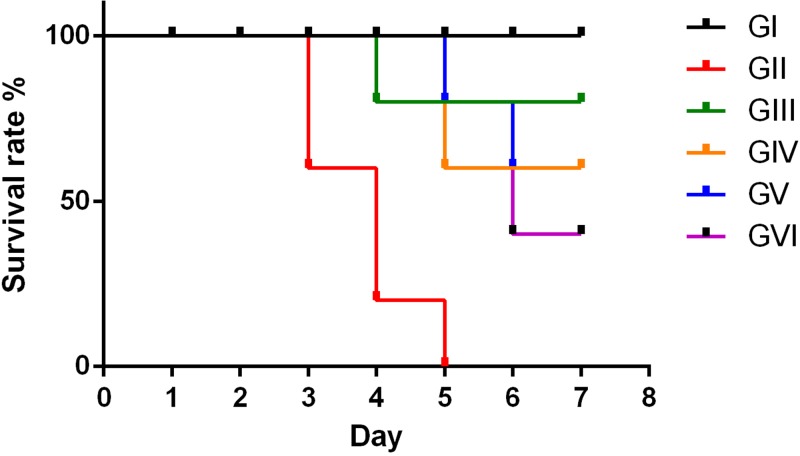
The survival rate among different groups studied in established toxoplasmosis (**GI**) Uninfected; (**GII**) Infected untreated; (**GIII**) Infected preventive dose with sulphadiazine; (**GIV**) Infected preventive dose with nanoparticles green synthesized with palm seeds and nabka treated; (**GV**) Infected treated with sulphadiazine; (**GVI**) Infected treated with nanoparticles green synthesized with palm seeds and nabka treated.

Clinically, the survival rate in infected untreated group (Group II) started in reduction on the third day post-infection and reduced progressively onwards, mostly died by the fifth day post infection. On the other hand, mice that were pretreated with sulphadiazine (group III) showed increase in survival rate that was 90% at the seventh day post infection (Figure 4). In case of AgNPs produced via date palm seeds and nabka extractions pretreated group (Group IV), there was a reduction on the third day post-infection and survival rate 70% by the seventh day post infection. However, the survival rate of mice was decreased when the sulphadiazine or AgNPs were administered post-infection. In this case, the survival rate in group V (drug post-infection group) was reduced on the third day and reduced progressively 60% at the seventh day post infection. Whereas AgNPs post-infection group, there was increased in the survival rate as compared with infected untreated control and 30% of the mice were remained at the seventh day post infection.

### Biochemical findings of liver functions

**Figure 5:**
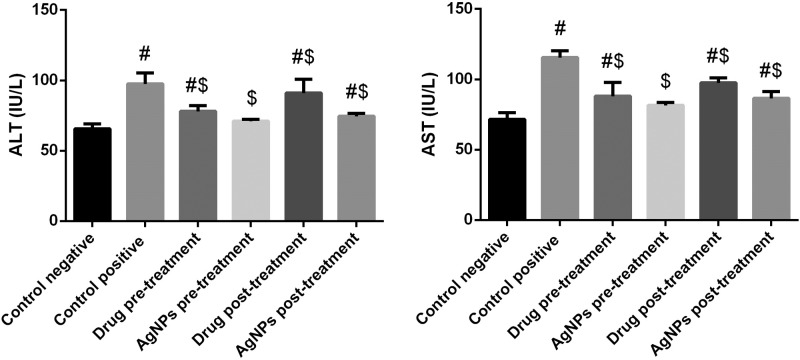
Histograms showing the effect of nanoparticles on plasma liver function markers of infected with *T. gondii* Values are the means ± SEM (*n*=7). #: significant change at *P*<0.05 with respect to the negative control group. $: significant change at *P*<0.05 with respect to the positive control group.

The infection by *T. gondii* caused hepatotoxicity that was indicated by the elevation of the activity of serum ALT and AST, whereas supplementation with AgNPs yielded a significant (*P*<0.05) decrease in the levels of marker enzymes and restored these to the control values as shown in Figure 5. In addition, plasma ALT and AST were significantly (*P*<0.05) decreased in the sulfadiazine groups (treated pre- and post-infection). Interestingly, our data indicate that AgNPs green synthesized with *P. dactylifera* and *Z. spina christi* were more effective than sulfadiazine in both treatment groups.

### Effect of AgNPs on lipid peroxidation (LPO)

**Figure 6:**
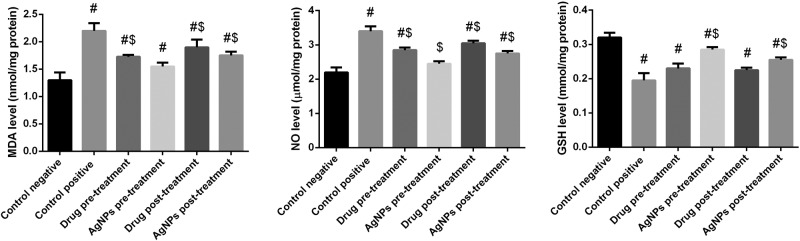
Histograms showing the effect of nanoparticles on hepatic oxidative stress markers of infected with *T. gondii* Values are the means ± SEM (*n*=7). #: significant change at *P*<0.05 with respect to the negative control group. $: significant change at *P*<0.05 with respect to the positive control group.

Lipid peroxidation (LPO) has been implicated in the pathogenesis of hepatic injury by the free radical derivatives of *T. gondii* and is responsible for cell membrane damage and consequent release of marker enzymes of hepatotoxicity. Malonaldehyde (MDA), an end product of lipid peroxidation, is widely used as a marker of LPO. *T. gondii* infection resulted in a significant increase in the hepatic MDA. Figure 6 showed that hepatic MDA was significantly increase in infected non-treated group level (*P*<0.05), while showed that significantly decrease in sulfadiazine groups (pre- and post-) (*P*<0.05) compared with the infected non-treated group. But this increase that inhibited in AgNPs groups (pre- and post-) (*P*<0.05). However, Ag nanoparticles green synthesized with *Phoenix dactylifera L* and *Zizyphus Spina-christi* are more effective than sulfadiazine groups.

### Effect of AgNPs on nitric oxide (NO)

*T. gondii* infection resulted in a significant increase in the levels of nitric oxide (NO) in liver homogenates. Figure 6 showed that hepatic NO was significantly increased in the infected non-treated group (*P*<0.05), whereas a significant decrease in sulfadiazine (pre- and post-) groups was observed (*P*<0.05) compared with the negative control group. Conversely, this increase was inhibited in the AgNPs groups (pre- and post-) (*P*<0.05). Moreover, our results demonstrate that AgNPs (*P. dactylifera* and *Z. spina christi*) were more effective than sulfadiazine in both treatment groups.

### Effect of AgNPs on glutathione (GSH)

*T. gondii* infection resulted in a significant decrease in the levels of GSH in liver homogenates. Figure 6 showed that hepatic GSH was significantly decreased in the infected non-treated group (*P*<0.05), and in sulfadiazine (pre- and post-) groups (*P*<0.05) compared with the negative control group. Conversely, this decrease was reversed in the AgNPs groups (pre- and post-) (*P*<0.05). Moreover, our results demonstrate that AgNPs (*P. dactylifera* and *Z. spina christi*) were more effective than sulfadiazine in both treatment groups.

### Effect of AgNPs on hepatic antioxidant enzymes (SOD)

**Figure 7:**
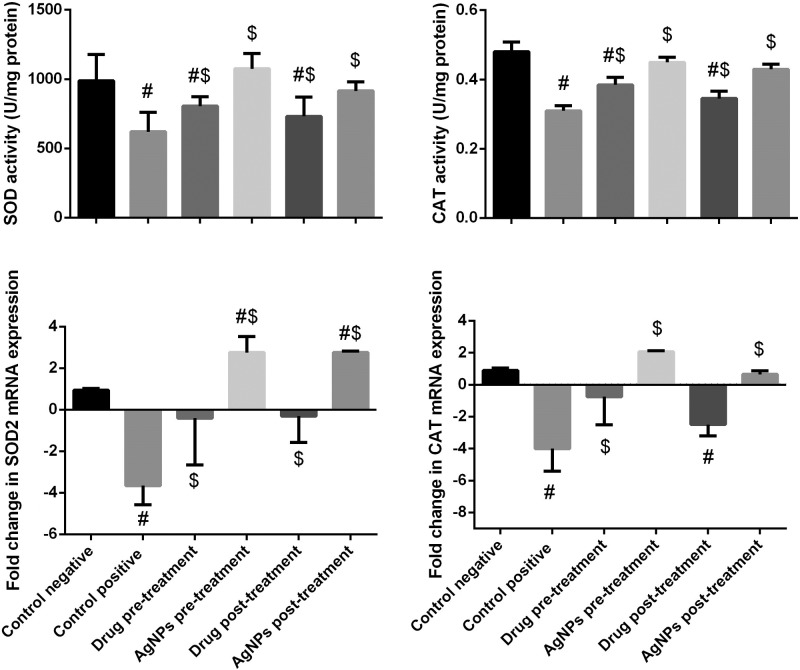
Histograms showing the effect of nanoparticles on antioxidant defense system of infected with *T. gondii* Values of biochemical results are the means ± SEM (*n*=7). Results for gene expression are presented as means ± SD of triplicate assays and normalized to β-actin and expressed as fold change (log2 scale), relative to mRNA levels in controls. #: significant change at *P*<0.05 with respect to the negative control group. $: significant change at *P*<0.05 with respect to the positive control group.

The effect of AgNPs on *T. gondii* induced a significant decrease in SOD functioning as an enzymatic antioxidant substance. Figure 7 showed that hepatic SOD was significantly decreased in the infected non-treated group, whereas a significantly increase can be observed in the sulfadiazine (pre- and post-) groups compared with the negative control group. Conversely, this decrease was inhibited in the AgNPs groups (pre- and post-). These results indicate that AgNPs (*P. dactylifera* and *Z. spina christi*) are more effective than sulfadiazine in both treatment groups.

### Effect of AgNPs on hepatic antioxidant enzymes (CAT)

The effect of AgNPs on *T. gondii* also induced a significant decrease in CAT. Figure 7 shows that hepatic CAT was significantly decreased in the infected non-treated group (*P*<0.05), whereas a significant increase in sulfadiazine (pre- and post-) groups can be observed (*P*<0.05) compared with the negative control group. Conversely, this decrease was inhibited in AgNP groups (pre- and post-). These data demonstrate that AgNPs (*P. dactylifera* and *Z. spina christi*) are more effective than sulfadiazine for both treatment groups.

### Effect of AgNPs on SOD2 and CAT mRNA expression by real time-PCR

#### Effect of AgNPs on *SOD2* mRNA

A significant down-regulation was observed in the gene expression levels of *SOD2* in the infected non-treated group and sulfadiazine group (pre- and post-) compared with the control group (Figure 7). In contrast, treatment with AgNPs (pre- and post-) caused a significant up-regulation in *Sod2* gene expression levels.

#### Effect of AgNPs on *CAT* mRNA

A significant down-regulation was observed in gene expression levels of *CAT* in the infected non-treated group and sulfadiazine groups (pre- and post-) compared with the control group (Figure 7). Conversely, treatment with AgNPs (pre- and post-) caused significantly up-regulated gene expression levels (*P*<0.05).

### Effect of AgNPs on proinflammatory cytokines

#### Proinflammatory cytokines levels

**Figure 8:**
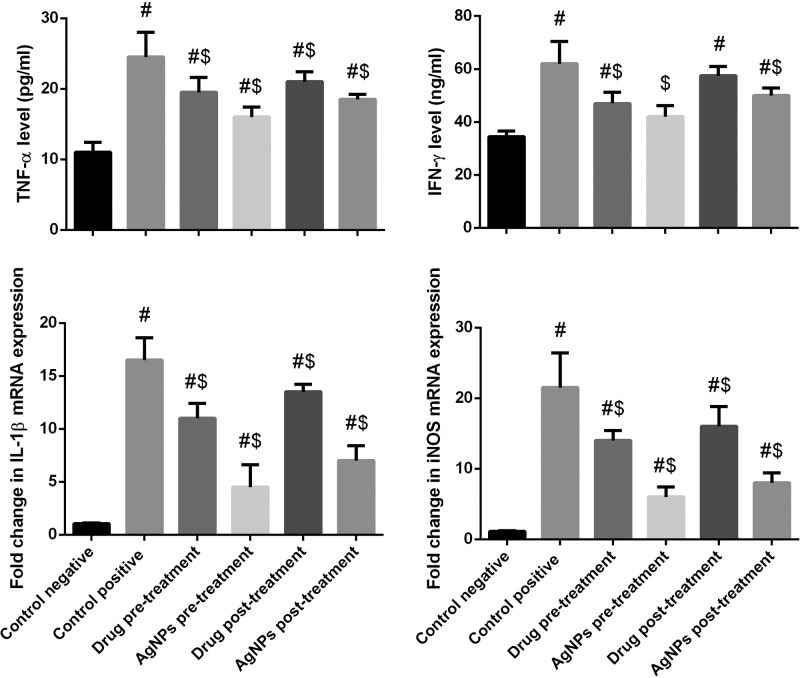
Histograms showing the effect of nanoparticles on proinflammatory cytokines of infected with *T. gondii* Values of biochemical results are the means ± SEM (*n*=7). Results for gene expression are presented as means ± SD of triplicate assays and normalized to β-actin and expressed as fold change (log2 scale), relative to mRNA levels in controls. #: significant change at *P*<0.05 with respect to the negative control group. $: significant change at *P*<0.05 with respect to the positive control group.

Inflammation is characterized by the release of proinflammatory cytokines such as TNF-α and IFN-γ. Figure 8 illustrated the effect of Ag nanoparticles on hepatic TNF-α and IFN-γ that showed significantly increase in infected non-treated group level (*P*<0.05), while showed that significantly decrease in sulfadiazine groups (pre- and post-) compared with the control group (-ve). But this increased that inhibited in Ag nanoparticles groups (pre- and post-). Moreover, Ag nanoparticles (Phoenix Dactylifera L & Zizyphus Spina Christi) are more effective than sulfadiazine groups.

#### Proinflammatory cytokines mRNA expression

The present study showed that a significant up-regulation in gene expression of iNOS mRNA and IL-1β mRNA that induced by *T. gondii* compared with the control group. In contrast, the treatment with Ag nanoparticles groups (pre- and post-) caused a significant decreased and down-regulation in gene expression levels of iNOS and IL-1β mRNAs (Figure 8).

#### Histopathological examinations

**Figure 9:**
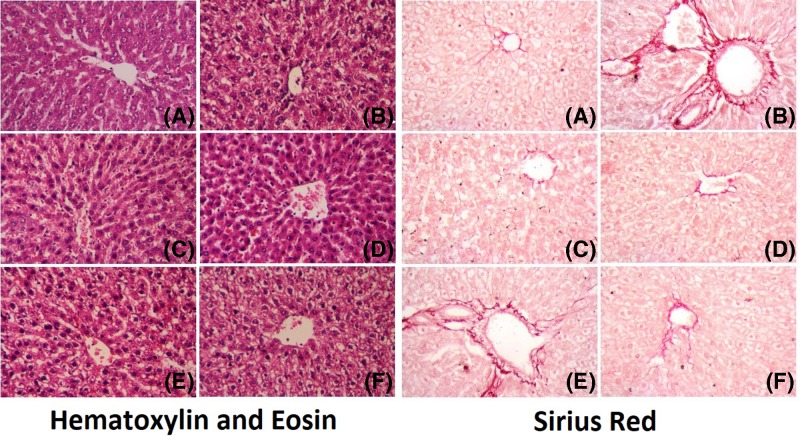
Photomicrographs for liver sections stained with hematoxylin and eosin or Sirius Red (**A**) Negative control section with normal hepatocytes architecture and low fibrosis, as evidenced by the deposition of collagen into areas surrounding the centrilobular veins, ×400. (**B**) Positive control section of the liver biopsy with severe liver injury and fibrosis, as evidenced by pericellular and periportal bridging fibrosis, ×400. (**C**) Sulfadiazine pretreated liver biopsy section with moderate fibrosis, as evidenced by the deposition of collagen into areas surrounding the centrilobular veins, ×400. (**D**) Sulfadiazine post-treated liver biopsy section with severe liver injury and fibrosis, as evidenced by pericellular and periportal bridging fibrosis, ×400. (**E**) Nanoparticle pretreated liver biopsy section with low fibrosis, as evidenced by deposition of collagen into areas surrounding the centrilobular veins. (**F**) Nanoparticle post-treated liver biopsy section with low fibrosis, as evidenced by deposition of collagen into areas surrounding the centrilobular veins, ×400.

Control animals showed no histopathological abnormality and low fibrosis, as evidenced by the deposition of collagen into areas surrounding the centrilobular veins (Figure 9A), whereas liver sections infected with *T. gondii* tachyzoites showed intensive architectural disarray with moderate fatty change and inflammatory cells infiltration and fibrosis, as evidenced by pericellular and periportal bridging fibrosis (Figure 9B). Sulfadiazine pretreated liver biopsy sections showed mild architectural disarray with *T. gondii* tachyzoites multiplying in the cells of the liver, mild fatty change and inflammatory cell infiltration and moderate fibrosis, as evidenced by the deposition of collagen into areas surrounding the centrilobular veins (Figure 9C), whereas the post-treated section showed intensive architectural disarray with *T. gondii* tachyzoites multiplying in the cells of the liver, heavy fatty change and inflammatory cell infiltration and severe fibrosis, as evidenced by pericellular and periportal bridging fibrosis (Figure 9D). Conversely, AgNPs pre-treated liver biopsy sections exhibited normal architectural with some inflammatory cell infiltration and low fibrosis, as evidenced by the deposition of collagen into areas surrounding the centrilobular veins (Figure 9E), whereas post-treated section showed intensive architectural disarray with *T. gondii* tachyzoites multiplying in the cell of the liver with apparent ameliorations, mild fatty change and inflammatory cell infiltration and low fibrosis, as evidenced by the deposition of collagen into areas surrounding the centrilobular veins (Figure 9F).

#### Immunohistochemistry for the detection of TGF-β and NF-κB

**Figure 10:**
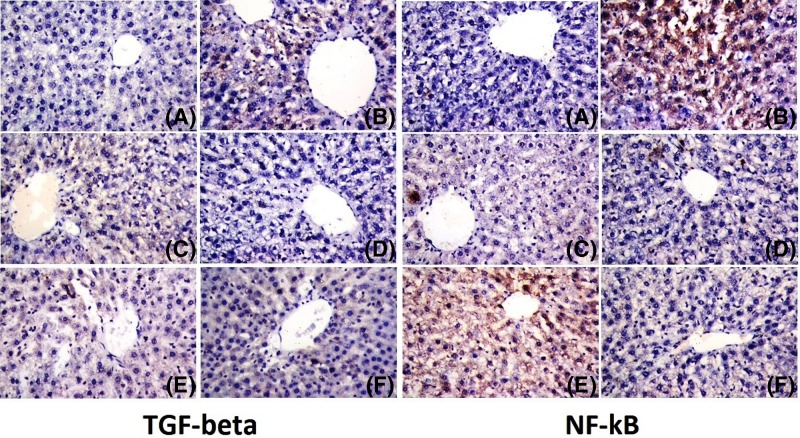
Histopathological examinations of the expression and specific tissue distribution of TGF-β and NF-κB (**A**) Liver sections from the control group with low TGF-β and NF-κB immune positive reaction in the hepatic cells, ×400. (**B**) Positive control section with moderate staining of TGF-β and NF-κB-positive hepatocytes around the central veins, ×400. (**C**) Sulfadiazine pretreated section with low staining of TGF-β and NF-κB-positive hepatocytes, ×400. (**D**) Sulfadiazine post-treated section similar to the pre-treated section with low staining of TGF-β and NF-κB-positive hepatocytes around the central veins, ×400. (**E**) Nanoparticle pretreated section with no staining of TGF-β and NF-κB-positive hepatocytes, 400×. (**F**) Nanoparticles post-treated section with low staining of TGF-β and NF-κB-positive hepatocytes, ×400.

Immunohistological study of the control liver displayed weak immunoreactivity to TGF-β and NF-κB in the hepatocytes as shown in Figure 10A. However, strong immunoreactivity to TGF-β and NF-κB-positive hepatocytes around the central veins was observed. Furthermore, livers challenged with *T. gondii* developed fibrosis and severe liver injury, as evidenced by periportal and pericellular fibrosis (Figure 10B). Conversely, sulfadiazine pre- (Figure 10C) and post-treatment (Figure 10D) induced a reduction in immunoreactivity to TGF-β and NF-κB in hepatic tissues. In addition, treatment by AgNPs also induced a reduction in immunoreactivity to TGF-β and NF-κB in hepatic tissues (Figure 10E,F). Furthermore, livers infected with *Toxoplasma* developed low fibrosis and injury, as evidenced by the deposition of collagen into areas surrounding the centrilobular veins. In this context, AgNPs treatment was more effective than chemotherapy.

## Discussion

Toxoplasmosis is a worldwide spread, water- and foodborne zoonotic ailment that caused by an unique protozoan *T. gondii* that infects all warm-blooded vertebrates including humans and birds [26]. Recent studies demonstrate that parasitic infections with high tolerance of the host are the result of defense mechanisms that include enhanced ROS generation. Oxidants and antioxidant biomarkers are known to exist under physiological circumstances. However, even small alterations in oxidant or/and antioxidant levels may disturb their balance and lead to oxidative injure. This condition becomes harmful when antioxidant system is incapable to restrain oxidative reactions triggered by ROS and directed oxidative modification of lipids, proteins and genomic DNA. Although free radicals are produced continuously by normal metabolic processes, their rate of production increase during certain parasitic infections. For example, stage switch between tachyzoite and bradyzoite forms of *Toxoplasma* is associated with morphological and molecular biological variabilities, including stage-specific antigen expression and alterations of host metabolism [27].

In addition, treatment of toxoplasmosis is often complicated owing to side toxic effects, cost and increase in drug resistance [28]. Such problems regarding toxoplasmosis treatment demand the continued effort to discover new improved anti-toxoplasmosis drugs. Notably, natural products have made and are continuing to make important contributions to the search for drugs in this category [29]. The use of plants as production assembly agents of AgNPs has drawn attention, because of the associated rapid, eco-friendly, non-pathogenic, and economical protocols and provision of a single step method for biosynthetic processes. Reduction and stabilization of silver ions is effected by the combination of biomolecules such as proteins, amino acids, enzymes, polysaccharides, alkaloids, tannins, phenolics, saponins and vitamins that are already established in plant extracts having medicinal values [30]. In addition to microbial organisms, plant extracts can also be used in the biosynthesis of metallic nanomaterials. For example *Ocimum basilicum*, *Camellia sinensis*, *Coriandrum sativum*, *Nelumbo nucifera*, *date palm*, *napka* and several other species have been studied as good source for the synthesis of AgNPs at a faster rate [31].

Date fruits have high compositions of carbohydrates, salts and minerals, dietary fiber, vitamins, fatty acids and amino acids, providing unique value in human nutrition and also playing a significant role in the neutralization of free radicals and finally suppressing the development and progression of various types of diseases. Earlier investigation by Al-Qarawi et al. [32] found that the palm date has a strong capability to suppress free radicals. Daoud et al. [33] reported that date fruits used in the management of diseases actively support the role of ROS in cancer and that dietary antioxidants as well as endogenous antioxidants showed a crucial role as preventive agents through the neutralization of ROS. Subsequently, Metwaly et al. [34] showed that plant phenolic compounds including flavonoids function as effective antioxidants with reported anti-mutagenic and anti-carcinogenic effects. Moreover, they are reported to possess a broad range of attractive properties, such as anti-fungal, anti-bacterial, anti-parasitic, anti-viral, anti-inflammatory and anti-apoptotic activities [35]. In particular, nabka (*Z. spina-christi*) species are frequently used in folklore medicine for the treatment of a variety of diseases such as different digestive disorders, general weakness, some liver complaints, severe obesity, diabetes, skin infections, loss of appetite, fever, pharyngitis, anemia and diarrhea [36,37]. Additionally, previous studies have shown that nabka has various therapeutic properties such as anti-bacterial, anti-diarrheal, anti-parasitic, anti-cancer, anti-fungal, anti-hyperglycemic, anti-inflammatory, anti-microbial, antioxidant, anti-diabetic and anti-tumor activities [38]. The curative effects of the extract may be due to the active components present such as alkaloids, flavonoids, saponins, proteins and lipids [39].

Liver diseases are among the most serious health problems in the world today and their prevention and treatment options remain limited despite the tremendous advances in modern medicine. The pathogenesis of hepatic diseases as well as role of oxidative injury and inflammation therein is well-established and, accordingly, blocking or retarding the chain reactions of oxidation and inflammation processes may comprise by promising therapeutic strategies for the prevention and treatment of liver injury [40]. The first sign of hepatic injury induced by toxoplasmosis can be obtained by the evaluation of ALT and AST, elevated levels of which recognized in cytotoxic and cholestatic hepatic injuries [41]. The reverse of these alterations by NPs serves as a clear indication of improvement of the functional status of hepatocytes along with preservation of cellular architecture indicating the hepato-protective activity of NPs [42]. Moreover, our results could highlight the mechanism underlying the AgNPs green synthesized by date palm and nabka hepatoprotective activity, being an antioxidant.

ROS formation during multiple normal processes in tissues and cells is indicative of the pathogenesis of various parasitic infections including *Leishmania* sp., *T. gondii*, *Giardia lamblia* and *Entamoeba histolytica* [43]. In the present study, *T. gondii* in acutely infected mice regulated ROS production in a manner inversely correlated with the degree of intracellular parasitization. Taken together, these findings suggest that both ROS-associated anti-*Toxoplasma* activity and parasite-driven suppression of ROS production underlie the pattern of ROS production [44]. Furthermore, NO radicals play an important role in inducing inflammatory response and their toxicity multiplies only when they react with O^2−^ radicals to form peroxynitrite that damages biomolecules [45]. Our results showed elevated levels of NO in toxoplasmosis inoculated mice consistent with its elevation that significantly decreased after treatment with AgNPs. This may therefore comprise a potential mechanism by which NPs can act as anti-inflammatory agents and thus protect the liver [46].

The overproduction of nitric oxide in response to the parasitic infection may be considered as one of the risk factors for inducing oxidative injury and inflicting tissue damage. Moreover, NO production correlates positively with tissue fibrosis by inducing fibrogenic cytokines and increasing collagen synthesis [47]. In our results, the NPs were active and may have served as highly potent and novel therapeutic agents for the quenching of NO. NPs may therefore also exert their effects on the regulation of pathological conditions caused by unwarranted generation of NO and its oxidation product-peroxynitrite. Notable, oxidative stress produced by free radicals is the main and primary step in toxoplasmosis toxicity contributing to both onset and progression of fibrosis [48]. This was evidenced by the enhanced lipid peroxidation, associated with decreased levels of the system of antioxidant enzymes; namely, SOD and CAT. It has been reported that antioxidant enzymes constitute a mutually supportive defense system against ROS. In the present study, we demonstrated that toxoplasmosis induced a significant decrease in the activities of antioxidant enzymes. This would cause an increased accumulation of superoxide radicals, which could further stimulate lipid peroxidation. The inhibition of antioxidant enzymes after toxoplasmosis is probably due to protein inactivation by ROS, as oxidative damage often leads to the loss of specific protein function [49]. SOD catalyzes the dismutation of superoxide anion to H_2_O_2_ and O_2_. Because H_2_O_2_ is still harmful to cells, catalase further catalyzes the decomposition of H_2_O_2_ to water. Thus, the coordinate actions of various cellular antioxidants in mammalian cells are critical for effectively detoxifying free radicals. Toxoplasmosis in mice diminished the antioxidant capacity of the mouse liver as evinced by decreased activity of the antioxidant enzymes, which is in agreement with others [49,50]. The positive effect of different classes of polyphenols and flavonoids on antioxidant enzyme activities *in vivo* has been shown in previous studies [51]. The results of SOD and CAT indicated that ROS production was highly elevated in the livers of mice after toxoplasmosis, thus further confirming that free radicals and oxidative damage certainly play a vital role in the pathogenesis of acute liver injury, and also providing strong support for the application of natural antioxidants in the treatment of toxic hepatopathy.

Glutathione is the most abundant non-protein thiol source in the cell, which acts as a substrate for several enzymes, including glutathione peroxidase [41]. In the present study, nanoparticles markedly increased and maintained the hepatic GSH level even after *T. gondi* infection. *T. gondii* leads to a significant depletion in the glutathione level that can be an important factor in the *T. gondii* toxicity. The mechanism of hepatoprotection by AgNPs against *T. gondii* toxicity might be due to restoration of the GSH level, date seed and nabka have the potential to be used as a supplement for antioxidants, and this is agreement with the previous reports [39,52].

Light microscopic examination of histological sections of the livers showed some changes produced by toxoplasmosis, such as necrosis, and infiltration of inflammatory cells amid intense architectural disorganization with *Toxoplasma* tachyzoites multiplying in hepatic cells. Conversely, following treatment with NPs, normal liver architectural was observed with limited inflammatory cell infiltration. Notably, *Toxoplasma* reproduction has not previously been observed in hepatic cells [53]. In the present study, NPs were able to partially restrain toxoplasmosis-induced decay of antioxidant enzyme activities; this preventive effect was also observed at the histological level. A similar scavenger role of flavonoids in mice and rats after exposure to toxoplasmosis had been determined in the liver [54]. The impairment of the antioxidant defense system is a critical step in toxoplasmosis-induced injury. At the gene expression level, the current findings indicated that, toxoplasmosis induced down-regulated expression of all examined antioxidant gene. Similar results were obtained in toxoplasmosis-induced liver fibrosis in mice [55]. The present findings also demonstrated that NPs augmented the antioxidant status through up-regulation of *CAT* and *SOD2* expression. Several studies have suggested that the phytochemical content and antioxidant/free radical scavenging effect of fruits and vegetables contribute to their protective effect against chronic and degenerative diseases [39]. Thus, we speculate that the protective effect detected in the present study is due to the antioxidant capability of NPs. Finally, the expression of TGF-β, considered a hallmark of fibrosis, increased during the onset of liver fibrogenesis. Immunohistochemical detection in the present study revealed high expression of TGF-β in the inoculated mice compared with that in the control group. It has been shown that general inhibitors of TGF-β could attenuate hepatic fibrosis or are useful to inhibit acute and chronic inflammatory or vascular diseases [56]. Our findings thus indicated that biosynthesized NPs might be considered potential chemotherapeutic agents affording high levels of safety and rapidity in the treatment of toxoplasmosis and therefore may constitute a possible new drug with anti-toxoplasmosis activity against *Toxoplasma*.

Nuclear factor-κB has a seminal role in immunity, because it activates proinflammatory genes encoding iNOS, COX-2, TNF-α, IL-1β and IL-6 [39]. It is activated by phosphorylation, ubiquitination and subsequent proteolytic degradation of the IκB protein by activated IκB kinase (IKK) [39]. The liberated NF-κB translocates to the nucleus and binds as a transcription factor to κB motifs in the promoters of target genes, leading to their transcription. Aberrant NF-κB activity is linked with various inflammatory diseases, and most anti-inflammatory drugs suppress inflammatory cytokine expression by inhibiting the NF-κB pathway [40]. Thus, an NF-κB inhibitor has clinical potential in inflammatory diseases. Our results in accordance Molestina et al. [57] with who found that *T. gondii* that might be used to manipulate the NF-κB signaling pathway in the host to elicit a survival response during infection. However, AgNPs suppressed NF-κB expression in liver tissue. The obtained results in agreement with Crisan et al. [58] who found that AgNPs sustained proinflammatory cytokines suppression from activated macrophages *in vitro* by decreasing phosphorylation of IκBα.

The interaction of host cells with *T. gondii* products or their invasion frequently results in IFN-γ- and TNF-α-producing CD4^+^ and CD8^+^ T cells activation and translocation of NF-κB. The results presented here reveal that *in vivo* infection with *T. gondii* results robust increase in proinflammatory cytokine levels and gene expression. During chronic *T. gondii* infection, lymphocytes play a pivotal role in parasite migration to the other peripheral organs by producing robust amounts of NO, IFN-γ and TNF-α. Indeed, the blockade of proinflammatory cytokines production prevented hypotension and improved the host survival upon sepsis induction [59]. However, AgNPs treatment was found to prevent inflammation and this effect may be due to the ability AgNPs in inhibiting NF-κB translocation to nucleus [14].

## Conclusions

In conclusion, the treatment with AgNPs (green synthesized by date palm and nabka) was more effective in inhibiting the development of hepatotoxicity than standard treatments through modulation of the cellular redox status, inflammation, up-regulation of antioxidant genes as well as improvement in the histological and immunohistological characteristics of the *T. gondii*-infected liver. These results suggest the novel therapeutic approaches of AgNPs green synthesized with date palm and nabka for intervention against the progressive liver damage associated with toxoplasmosis.

## Abbreviations

CAT: catalase
GSH: glutathione
IKK: IκB kinase
LPO: lipid peroxidation
PBS: phosphate-buffered saline
NO: nitric oxide
NP: nanoparticle
SOD: superoxide dismutase
